# Is Supersize More than Just Too Much Food?

**DOI:** 10.1289/ehp.1205200

**Published:** 2012-06-01

**Authors:** Linda S. Birnbaum

**Affiliations:** Director, NIEHS and NTP, National Institutes of Health, Department of Health and Human Services, Research Triangle Park, North Carolina, E-mail: birnbaumls@niehs.nih.gov

Diabetes and obesity are two of the most significant public health issues of our day, and both are major epidemics in the United States and abroad. These conditions are interrelated; obesity has been long recognized as a common precursor to adult-onset (type 2) diabetes, although “adult-onset” is becoming an outdated term. In the United States, the prevalence of obesity among children and adolescents has almost tripled since 1980, and an estimated 12.5 million children and adolescents (16.9%) are considered obese (Ogden and Carroll 2010). This trend is also apparent in preschool children 2–5 years of age, a group in which obesity increased from 5% in 1976–1980 to 10.4% in 2007–2008 (Ogden and Carroll 2010). One report based on well-child visits at a health maintenance organization in Massachusetts was particularly disturbing: The prevalence of overweight in infants 0–6 months of age almost doubled between 1980 and 2001, from 3.4% to 5.9% (Kim et al. 2006). This finding suggests that factors other than changes in physical activity or diet are contributing to these trends, pointing to possible changes in fetal programming.

The most recent estimates of diabetes prevalence in the United States are equally staggering. Based on data from 2005 through 2008, 25.6 million (11.3%) of all people in the United States ≥ 20 years of age have diagnosed or undiagnosed diabetes (Centers for Disease Control and Prevention 2011). Another 35% have prediabetes, a condition in which blood glucose is higher than normal but not high enough to be classified as diabetes. People with prediabetes have an increased risk of developing type 2 diabetes, heart disease, and stroke.

Being overweight or obese has been estimated to account for approximately 70% of the cases of type 2 diabetes (Eyre et al. 2004). However, the etiology of the remaining 30% is unknown. Given the sheer numbers of people with the disease—now estimated globally at 220 million and expected to grow to 366 million by 2030 (World Health Organization 2011)—it is easy to understand the growing consideration of “nontraditional” risk factors (e.g., environmental chemicals, stress, microbiome) as contributors to these diseases. A growing scientific literature implicating a role for environmental chemical exposures has been developed largely through the funding of the National Institute of Environmental Health Sciences (NIEHS) as part of the institute’s broader interest in understanding endocrine-related disorders and the developmental origins of adult disease. Endocrine-disrupting chemicals alter control of adipose tissue development and function, control of food intake, insulin sensitivity, glucose homeostasis, and lipid metabolism (Janesick and Blumberg 2011; Nadal et al. 2009; Thayer et al. 2012). If the exposure occurs during development, the result could possibly be an altered “set point” or sensitivity for developing obesity or diabetes later in life.

Research addressing the role of environmental chemicals in diabetes and obesity has rapidly expanded in the past several years. Both the May 2010 White House Task Force on Childhood Obesity (2010) and the 31 March 2011 *Strategic Plan for NIH Obesity Research* [NIH (National Institutes of Health) Obesity Research Task Force 2011] acknowledge the growing science base in this area and cite the need to understand more about the role of environmental exposures as part of future research and prevention strategies.

To help develop such a research strategy, the National Toxicology Program (NTP), with collaboration from the NIEHS intramural and extramural program scientists, organized a state-of-the-science workshop in January 2011 titled “Role of Environmental Chemicals in the Development of Diabetes and Obesity.” The technical background documents assembled for this workshop were extensive, totaling > 500 pages and spanning the range from epidemiological data to high throughput screening results. As an additional scientific resource, approximately 800 main findings from the epidemiological studies of diabetes and childhood obesity have been compiled into a searchable graphing software program. A diverse group of > 150 scientists, including toxicologists, epidemiologists, and bioinformaticists, as well as experts in the pathobiology of diabetes and obesity, attended the meeting to review the existing literature and shape a research strategy.

The review of the collected literature supported the plausibility of certain environmental chemicals acting as “obesogens” or diabetogenic agents. In some cases, the conclusions were based on surprisingly consistent epidemiological associations. With other chemicals or chemical classes, consistency was found in mechanisms of action. We have little appreciation for the extent to which environmental chemical exposures may be influencing obesity and diabetes rates, but it is becoming increasingly clear that overnutrition and a lack of exercise are not the entire story.

The first of a series of articles stemming from the January 2011 workshop appears in this issue of *Environmental Health Perspectives* (Thayer et al. 2012). Kristina Thayer, director of the NTP Office of Health Assessment and Translation, other NIEHS staff, and the workshop chair, Michael Gallo (University of Medicine and Dentistry of New Jersey–Robert Wood Johnson Medical School) provide an introduction to the topic and an orientation to the workshop and key outcomes. Upcoming reports will examine the influence of smoking during pregnancy, as well as nicotine and arsenic exposures, on diabetes and obesity outcomes and mechanisms.
